# Mechanistic insights into drying methods: how they govern the structure and bioactivity of resveratrol-HP-β-CD inclusion complexes

**DOI:** 10.3389/fchem.2025.1692674

**Published:** 2025-12-19

**Authors:** Adilah Marwa, Anton Bahtiar, Ahmad Fuad Shamsuddin, Ariawan Gunadi, Witta Kartika Restu, Mahdi Jufri

**Affiliations:** 1 Faculty of Pharmacy, University of Indonesia, Depok, West Java, Indonesia; 2 Department of Pharmacy, Institute of Tarumanagara, South Jakarta, Indonesia; 3 Faculty of Pharmacy and Health Sciences, Royal College of Medicine Perak, Universiti Kuala Lumpur (RCMP UniKL), Ipoh, Perak, Malaysia; 4 Research Center for Chemistry, National Research and Innovation Agency (BRIN), Serpong, Indonesia

**Keywords:** freeze dryer, hydroxypropyl-β-cyclodextrin, inclusion complex, resveratrol, solvent evaporation, spray dryer

## Abstract

Resveratrol (RES), a polyphenol with notable therapeutic potential, faces clinical limitations due to poor water solubility and low bioavailability. This study aimed to enhance the solubility and stability of RES by forming inclusion complexes with hydroxypropyl-β-cyclodextrin (HP-β-CD) using three drying methods: spray drying (SD), freeze-drying (FD), and solvent evaporation (SE). Phase solubility analysis confirmed the successful formation of a stable complex (K = 5278 M^-1^) at an optimal 1:2 RES: HP-β-CD ratio. Among the dried products, the spray-dried complex (RHSD) demonstrated superior performance in moisture content, yield, and resveratrol content. It achieved the highest solubility enhancement (89-fold increase). In contrast, the freeze-dried complex (RHFD) showed the most potent antioxidant activity (IC_50_ = 14.01 μg/mL). Physicochemical characterization via FTIR confirmed the formation of the inclusion complex. At the same time, XRD and SEM analyses revealed that RHSD possessed an amorphous structure, whereas RHFD and RHSE exhibited semi-crystalline characteristics. Subsequent drug-release studies demonstrated that RHSD followed non-Fickian release kinetics, unlike the diffusion-controlled release of RHFD and RHSE. These findings demonstrate that spray drying produces complexes with optimal physicochemical properties for solubility and dissolution, while freeze drying better preserves antioxidant activity, providing crucial insights for developing resveratrol-cyclodextrin formulations in pharmaceutical applications.

## 1 Introduction

Resveratrol (RES), a naturally occurring polyphenolic compound, has garnered considerable scientific interest due to its diverse biological activities, including potent antioxidant properties (Sadi and Konat, 2016), anti-inflammatory effects (Dull et al., 2019), and cardioprotective effects (Bostancıeri et al., 2022). Found primarily in the skins of grapes, berries, and certain nuts, resveratrol has been extensively studied for its potential therapeutic applications (Mohammadshahi et al., 2014; Pannu and Bhatnagar, 2019; Wang et al., 2017). Despite its promising therapeutic benefits, the clinical application of resveratrol is significantly hindered by its inherent physicochemical properties, such as poor aqueous solubility (Log P = 3.1), rapid metabolism, and low bioavailability (Berman et al., 2017). This critical gap between *in vitro* efficacy and *in vivo* performance is unequivocally demonstrated by multiple pharmacokinetic studies. Specifically, an *in vivo* study by Peñalva et al. (2018) revealed that even a substantial dose of 15 mg/kg in rats yielded only an average plasma concentration of 0.96 µM. This pattern is consistently observed in human clinical trials, where clinical doses of 1000 mg and 500 mg resulted in average concentrations of merely 0.79 µM and 0.312 µM, respectively (Kemper et al., 2022; Sergides et al., 2016). These values fall drastically short of the established effective concentration range of 5–10 µM required for resveratrol’s pharmacological activity *in vitro* (Ornstrup et al., 2016; Singh and Pai, 2014; Zhao et al., 2019; Zu et al., 2016). This pronounced disparity directly highlights how resveratrol’s physicochemical properties significantly limit its pharmacological effectiveness. These limitations have led to a growing interest in the development of innovative delivery systems that can enhance the solubility, stability, and bioavailability of resveratrol, thereby improving its efficacy in therapeutic applications (Balata et al., 2016; Calvo Castro et al., 2018; Dhakar et al., 2019).

**FIGURE 1:**
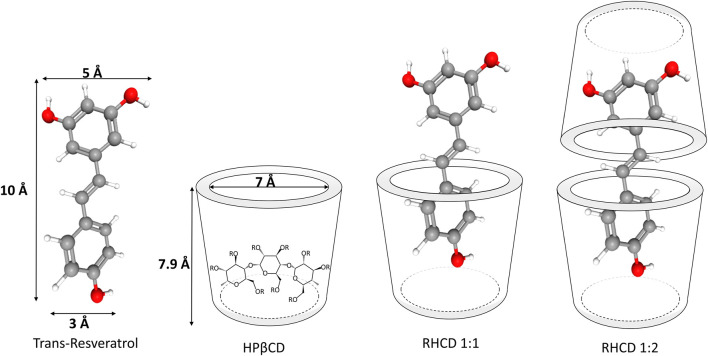
The molecular structure of chosen *trans*-RES and HP-β-CD.

One of the most promising strategies to overcome these challenges is the formation of inclusion complexes with cyclodextrins (Saokham et al., 2018). Cyclodextrins are a class of cyclic oligosaccharides well known for improving the solubility and stability of poorly soluble compounds. Among the different types of cyclodextrins, hydroxypropyl-β-cyclodextrin (HP-β-CD) has emerged as a particularly effective host molecule due to its excellent water solubility, biocompatibility, and low toxicity (Hao et al., 2021; Yergey et al., 2017). HP-β-CD has a unique molecular structure, characterized by a hydrophobic central cavity and a hydrophilic exterior Figure 1 enabling it to form stable inclusion complexes with lipophilic molecules such as resveratrol through host-guest interactions (Yang et al., 2022). Encapsulation of resveratrol in the HP-β-CD cavity is a promising approach for pharmaceutical and nutraceutical applications, as it can significantly increase its solubility, protect it from degradation, and improve its bioavailability (Saokham et al., 2018). As performed by Yang et al., development of HP-β-CD microcapsules for co-encapsulation of resveratrol significantly increased solubility by approximately 18-fold using spray drying methods.

The preparation of resveratrol inclusion complexes with HP-β-CD involves various techniques, each with distinct advantages and limitations. The supercritical antisolvent method has been proven to form a complex between resveratrol and HP-β-CD, resulting in a 25-fold increase in solubility (Zhou et al., 2012). Previous research conducted by Zhou et al., reported a significant increase in the solubility of resveratrol when complexed with steviol glycosides (STE) and hydroxypropyl-β-cyclodextrin (HP-β-CD) using different preparation methods, including ball and jet milling. The solubility of resveratrol was increased up to 30-fold after complexation. However, these methods still use high amounts of organic solvents and expensive equipment. This emphasizes that the choice of preparation method is very important as it affects the physico-chemical characteristic, encapsulation efficiency, and dissolution behavior of the resulting complexes (Saokham et al., 2018). There are other more commonly used methods for complexation process such as spray drying (15–18), freeze drying [17,18] and solvent evaporation (Zhou et al., 2012), which offer different advantages and limitations that affect the properties of the final product. Spray drying is widely recognized for its ability to produce fine, uniform particles, resulted in increased solubility. However, it requires high temperatures which can lead to degradation of heat sensitive compounds (Yang et al., 2022). Meanwhile, solvent evaporation is a milder method that operates at lower temperatures compared to spray drying, generally producing highly stable crystalline or semi-crystalline complexes, but tends to produce complexes with larger particle sizes (Sarabia-Vallejo et al., 2023). In contrast, lyophilization preserves the bioactivity of compounds by operating at low temperatures and creating amorphous structures which may increase the dissolution rate. However, this process is time-consuming and can result in larger diameters and less uniform particles (Wilkowska et al., 2016). Despite extensive research on methods creating resveratrol-cyclodextrin complexes, a direct comparison and highlight the effects of these techniques on the solubility, stability, and structural integrity of the final product remains absent. This comparison is crucial to identify the most efficient and cost-effective method for optimizing the resveratrol’s therapeutic potential.

This study address the knowledge gap by comparing the effects of spray drying (SD), lyophilization (FD) solvent, and evaporation (SE) on resveratrol complexes with HP-β-CD physical and functional properties. This study assesses each method’s advantages and drawbacks with particular attention to the resveratrol complex’s solubility, antioxidant activity, release profile, stability, morphology, and thermal properties. These research findings aim to improve our understanding of resveratrol complex preparation, leading to more effective resveratrol complex formulations, thereby enhancing resveratrol therapeutic potential and expanding its nutraceutical and pharmaceutical industries applications.

## 2 Materials and methods

### 2.1 Materials

Xi’an Natural Field Bio-Tech, China supplied resveratrol pharmaceutical grade (purity >98%). HP-β-cyclodextrin (purity >98%) was obtained from Xi’an Demeter Biotech Co., Ltd., China. Aquabidest was obtained from Ikapharmindo, Indonesia. HPLC-grade acetonitrile and methanol were supplied by Merck KGaA, Germany.

### 2.2 Methods

#### 2.2.1 Phase solubility study of resveratrol and cyclodextrin

The phase solubility study of the Resveratrol-HP-β-CD inclusion complex was done following the Higuchi and Connors method (Sarabia-Vallejo et al., 2023). Excess resveratrol (925 mg) was added to 10 mL phosphate buffer saline (PBSpH 7.4) containing 0–15 mM/L HP-β-CD and stirred at 25 °C for 24 h. After 24 h, the solution was filtered through a 0.45 µm PTFE membrane filter, and the resveratrol content was quantified by HPLC. The phase solubility diagram was obtained by plotting resveratrol solubility against HP-β-CD concentration. Equation 1 was used to calculate the apparent stability constant (Kc) of the Resveratrol:HP-β-CD inclusion complexes from the slope of the phase solubility diagram and the solubility of inclusion complex in the absence of CD (S0).

$$Kc=\frac{Slope}{S0\;\left(1-Slope\right)}$$ (1)

#### 2.2.2 Determination stoichiometry resveratrol-HP-β-CD complex

The stoichiometry of the Resveratrol-HP-β-CD inclusion complex was determined using the continuous variation method proposed by Job (1928). This method is used to establish the optimal molar ratio of host-guest interactions by analyzing changes in physicochemical properties as a function of composition variation. This method utilized UV-Vis spectrophotometry to measure complexation-induced absorbance changes at 306 nm, the maximum absorption wavelength of resveratrol. The Job plot, constructed by plotting ΔA × R against R, where ΔA represents the difference in resveratrol absorbance with and without HP-β-CD, and R is the ratio [Resveratrol]/([Resveratrol] + [HP-β-CD]), with the HP-β-CD fraction varied from 0.1 to 0.9.

#### 2.2.3 Preparation method of inclusion complex

**FIGURE 2:**
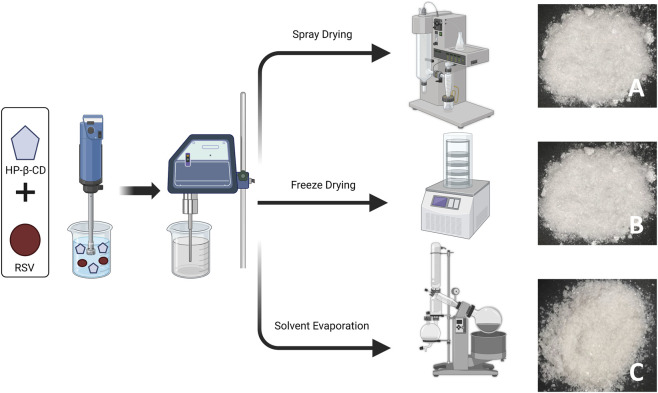
Preparation process and finished product diagram **(A)** RHSD 1:2; **(B)** RHFD 1:2; **(C)** RHSE 1:2.

Complexes of resveratrol and HP-β-CD were prepared in molar ratios of 1:1 and 1:2 according to the molecular weights of the two substances (Yang et al., 2022). Resveratrol (228 g/mol) was dissolved in 30 mL of water and HP-β-CD (1540 g/mol) was dissolved in 70 mL of water. Homogenizer (Ultraturrax T-25 Easy Clean, Germany) was used to mix both solutions at 14,000 rpm for 10 min, followed by ultrasonication (Qsonica CL-334, United States) for 10 min (pulse ON 3 s and pulse OFF 2 s) at 55% amplitude. The resulting homogeneous dispersion was then dried using a rotary evaporator (Buchi Rotavapor® R-300, Flawil, Switzerland), a spray dryer (Buchi Mini Spray Dryer B-290, Flawil, Switzerland), and a freeze dryer (Labconco FreeZone 6 L, Labconco Corporation, MO, United States). The drying conditions for the three tools are shown in the Table 1; Figure 2.

**TABLE 1:** Drying conditions using rotary evaporator, spray dryer, and freeze dryer.

Rotary evaporator (RHSE)	Spray dryer (RHSD)	Freeze dryer (RHFD)
Temperature: 61 °C Pressure: 61 mbar Rotation speed: 60 rpm Time: 2 h	Inlet temperature: 180 °C Outlet temperature: 125 °C Pump: 0,07% Aspirator: 85%	Temperature: −80 °C Time: 72 h

#### 2.2.4 Moisture content

Mettler Toledo HB43-S Compact Halogen Moisture Analyzer was used to quantify the moisture content of inclusion complex powder. 0.5 ± 0.005 g samples were placed on an aluminum plate and then heated at 105 °C. The moisture analysis was done in three replication measurements (Nguyen and Yoshii, 2018).

#### 2.2.5 Percent yield

The percentage yield (PY) is the ratio of the final weight (drug and polymers) after drying to the initial weight before complexation, and was calculated using Equation 2.

$$PY\;\left(\%\right)=\frac{Practical\;Mass\;\left(Inclusion\;complex\right)}{Theoritical\;Mass\;\left(Drug+Carrier\right)}\;x\;100$$ (2)

#### 2.2.6 Drug content

The determination of resveratrol content in inclusion complex powder was conducted using HPLC with a reversed-phase Zorbax Eclipse Plus C18 column (250 × 4.6 mm, 5 μm). The solution was filtered using a 0.45 μm PTFE membrane, before 20 μL was injected into the HPLC system. Detection was performed using a UV-Vis detector at 306 nm, the maximum absorption wavelength of resveratrol (Jagwani et al., 2020). The analysis was conducted using an isocratic elution method with a mobile phase of Acetonitrile:Deionized water (30:70, v/v). This composition was selected to ensure optimal solubility and peak resolution for resveratrol. The flow rate was set at 1.0 mL/min.

#### 2.2.7 Saturated solubility

Excess resveratrol and RHSD, RHFD, and RHSE inclusion complexes were added. Then, 10 mL of phosphate buffer pH 7.4 was added and stirred continuously for 24 h. After 24 h of stirring, 1 mL of the solution was removed by centrifugation at 13,000 rpm for 15 min at 4 °C. The supernatant was then collected and measured by HPLC. The HPLC analysis was performed under the following conditions a Zorbax Eclipse Plus C18 column (250 × 4.6 mm, 5 μm), a mobile phase of acetonitrile: deionized water (30:70, v/v) at a flow rate of 1.0 mL/min, and UV detection at 306 nm. The injection volume was 20 μL, and an isocratic elution mode was employed to ensure accurate and reliable quantification of resveratrol.

#### 2.2.8 Characterization of antioxidant activity

The antioxidant activity was evaluated using a modified method based on a previous study (Buljeta et al., 2022). Initially, resveratrol (RES) and its inclusion complexes (RHSD, RHSE, RHFD) were dissolved in ethanol to prepare solutions with concentrations ranging from 0 to 100 μg/mL. Subsequently, 1 mL of the sample solution was mixed with 1 mL of 250 μg/mL DPPH ethanol solution and stored in the dark for 30 min. After the incubation period, the mixture was centrifuged at 4000 g for 5 min. The absorbance of the obtained supernatant was measured at 517 nm using a UV-Vis spectrophotometer (UV-2600, Shimadzu, Tokyo, Japan). Measurement of blank treatment absorbance without any samples was also performed.

#### 2.2.9 Functional group analysis

FT-IR spectrometer (Bruker-Tensor II, Germany) were used to measured FT-IR spectra of resveratrol, HP-β-CD, RHSD, RHFD, and RHSE (1:2) inclusion complexes in scanning range of 4000–500 cm^-1^ of wavenumber. Each sample was mixed with KBr powder before applied to the spectrometer (Yang et al., 2022).

#### 2.2.10 Crystalline structure analysis

X-ray diffractometer (Rigaku, Japan) was used to obtain the diffraction patterns of the samples with Cu Kα radiation. The used current was 30 mA and the voltage was 40 kV. The sample measured on diffraction angle at 2 θ in the range of 10-45^O^, scanning speed at 1.5^o^/min, and step size of 0.05.

#### 2.2.11 Thermal characterization

Thermal analysis was conducted using a DSC system (STA 449F3, NETZSCH, Germany) under a dynamic nitrogen atmosphere (50 mL/min). Prior to analysis, the aluminum crucible was meticulously cleaned with methanol and dried at 80 °C. Approximately 5 mg of each sample was uniformly placed into the crucible and hermetically sealed. The temperature program included an initial stabilization at 25 °C for 5 min, followed by heating from 25 °C to 300 °C at a rate of 10 °C/min, and subsequent cooling back to 25 °C at the same rate. Both heating and cooling cycles were recorded to comprehensively analyze thermal behavior. The instrument was calibrated using indium standards, and all experiments were performed in triplicate to ensure reproducibility.

#### 2.2.12 Particle morphology analysis

SEM (JEOL JSM-IT200, Japan) were used to obtain the surface morphology of RHSD, RHFD, and RHSE (1:2) inclusion complexes by manually dispersing each sample in the conductive adhesive attached to the aluminum sheet.

#### 2.2.13 *In vitro* release studies

In this study, a dialysis membrane (Spectra/Por 4, molecular cut-off 12-14 kD, California, CA, United States) soaked in phosphate buffer saline (PBS) for 60 min was loaded with 1.5 mg samples of resveratrol and RHSD, RHFD, RHSE inclusion complex powder. The dialysis membrane was incubated at 37 °C (IKA® RT10) at 100 rpm in 150 mL of a dialysis solution of 0.05% tween 80 (v/v) in PBS pH 7.4. At predetermined intervals of 0.25 h, 0.5 h, 0.75 h, 1 h, and 6.5 h, 1 mL was removed from the dialysis bag and diluted 1:1 (v/v) with methanol. Quantification of resveratrol was performed using HPLC methods. *4.2.5* Each release test were performed in triplicate to ensure reproducibility.

The kinetics of the resveratrol released from inclusion complexes were determined by fitting the release profiles to the Zero order (Equation 3), First order (Equation 4), Korsmeyer–Peppas (Equation 5), and Higuchi (Equation 6) theoretical models:

$$F_{t}=K_{0}.t$$ (3)

where $F_{t}$ represents the fraction of drug released at time t , and $K_{0}$ is the zero-order release constant. This model assumes a constant release rate, independent of drug concentration.

$$F_{t}=1\;–\;\exp^{\left(-K1.t\right)}$$ (4)

where F_t_ represents the fraction of resveratrol released at time *t*, and *K*_*1*_ is the first-order release constant.

$$\frac{Mt}{Mi}=K_{kp}.t^{n}$$ (5)

where *M*_*t*_ */M*_*i*_ is the fraction of drug released into the release medium, M_t_ is the cumulative release at time *t*, and *M*_*i*_ is the initial amount of drug. *K*_*kp*_ is the Krosmeyer-Peppas constant, and *n* is the release exponent, which indicates the mechanism of drug release.

$$F_{t}=K_{H}t^{0.5}+a$$ (6)

where K_H_ is the Higuchi release constant, and a represents the initial drug release characteristic.

The zero order, first order, Korsmeyer-Peppas (KP), and Higuchi models were selected based on their established applicability for analyzing drug release from polymeric and matrix systems (Gasner et al., 2025; Ghizdovat et al., 2025; Zhang et al., 2021). The best-fitting kinetic model was selected based on the coefficient of determination (R^2^) and the release exponent (*n*) from the Korsmeyer–Peppas equation. A model with an R^2^ value closest to 1 was considered the most appropriate to describe the release behavior, whereas the *n* value provided additional insight into the mechanism of drug release, distinguishing between Fickian, non-Fickian, and Case-II transport processes. Additionally, the Model Selection Criterion (MSC) was used to evaluate model efficiency, balancing goodness of fit and model complexity, ensuring the selection of the most appropriate model for the release kinetics.

#### 2.2.14 Statistical analysis

The assay test, yield percentage, and moisture content result were analyzed statistically in comparison with the control group. Normality and homogeneity test were performed initially to determine the statistical method used. The normally distributed and homogenous data analysis was performed by one-way ANOVA (p < 0.05) followed by a *post hoc* test using the Tukey test. The Kruskal-Wallis test was carried out when the data was not normally distributed and the analysis continued with the Mann-Whitney test.

## 3 Result and discussion

### 3.1 Phase-solubility study of resveratrol

**FIGURE 3:**
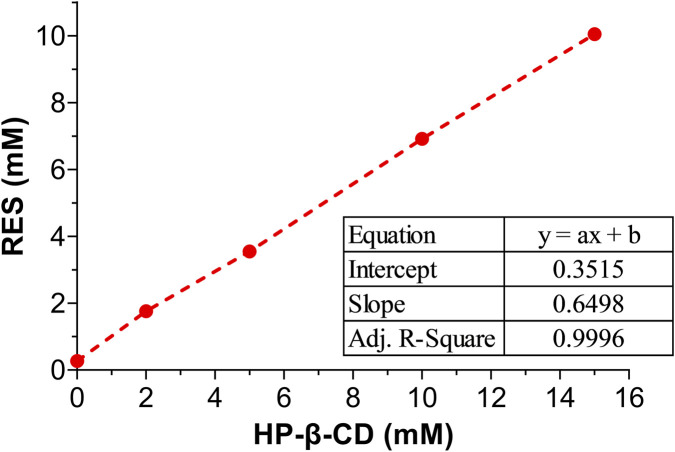
The phase-solubility diagram of resveratrol and HP-β-CD.

The inclusion of resveratrol with HP-β-CD significantly enhances its aqueous solubility, as demonstrated in the phase solubility plot depicted in Figure 3. The linear correlation between the solubilized resveratrol concentration and HP-β-CD concentration indicates an Aqueous Solubility Type L (AL-type) profile (Ulatowski et al., 2016). AL-type curves suggest that as the HP-β-CD concentration increases, resveratrol’s solubility also increases proportionally, without forming higher-order complexes. This behavior is consistent with previous studies on polyphenol-cyclodextrin interactions, where the solubility enhancement is linear and does not result in the formation of more complex structures (Shen et al., 2020).

The stability constant (Kc) of 5278 M^-1^ at 298 K indicates a strong binding affinity between resveratrol and HP-β-CD. According to Higuchi and Connors’ phase solubility classification, a stability constant (Kc) in the range of 1000–5000 M^-1^ is generally considered optimal for improving drug solubility while maintaining efficient drug release (Saokham et al., 2018). The high Kc value in this study suggests an effective complexation, ensuring that resveratrol remains in solution while being sufficiently released under physiological conditions (Sarabia-Vallejo et al., 2023; Shah et al., 2021). This result is consistent with previous reports on phenolic compound-cyclodextrin inclusion complexes, where the encapsulation of quercetin (Wangsawangrung et al., 2022), curcumin (Nikolić et al., 2023), and oxyresveratrol (He et al., 2019) with HP-β-CD demonstrated improved aqueous solubility and stability. The complexation stability is primarily driven by hydrogen bonding, van der Waals forces, and hydrophobic interactions between the aromatic rings of resveratrol and the nonpolar cavity of HP-β-CD (Iskineyeva et al., 2022). The detailed calculations supporting these findings can be found in Supplementary Appendix 1.

### 3.2 Determination of resveratrol inclusion complex stoichiometry

**FIGURE 4:**
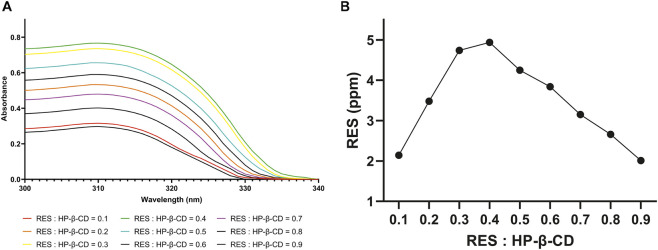
**(A)** UV spectra of aqueous solution of resveratrol and HP-β-CD mixed in variable molar ratio R; **(B)** Job’s plot different molar ratios of resveratrol and HP-β-CD from absorbance 306 nm measurements.

This study determined the stoichiometric ratio of the inclusion complex between resveratrol and hydroxypropyl-β-cyclodextrin (HP-β-CD) using the Job’s plot method, as shown in Figure 4. Figure 4A presents the UV-Vis spectra of various molar ratios of resvertarol: HP-β-CD (0.1–0.9), demonstrating systematic changes in absorbance, which indicate the formation of inclusion complexes at different ratios. The detailed results of absorbance and the calculation of the Job’s plot are provided in the Supplementary Appendix 2. The peak of the absorbance changes was further analyzed through the Job’s plot curve depicted in Figure 4B. As shown in Figure 4B, the maximum point of the curve was observed at an R-value of 0.4. According to the classical Job’s method theory, an R-value of 0.5 suggests a 1:1 complex, while an R-value of 0.33 suggests a 1:2 complex. Thus, the obtained R-value of 0.4 lies between these two ratios, suggesting a shift towards forming a 1:2 complex.

Referring to the theory proposed by Ulatowski et al. (2016), a peak in Job’s plot that deviates from the ideal 0.5 may indicate the coexistence of multiple types of complexes in solution, specifically a dynamic equilibrium between 1:1 and 1:2 complexes. In other words, at lower concentrations, a 1:1 complex is likely to form initially, while at higher concentrations, additional interactions may promote the formation of a 1:2 complex (Ulatowski et al., 2016). Based on these findings, it can be concluded that the resveratrol: HP-β-CD system exhibits a dynamic equilibrium between 1:1 and 1:2 inclusion complexes. Therefore, further analysis of each stoichiometric ratio was performed separately to achieve a more accurate interpretation of the data.

### 3.3 Evaluation of optimum ratio for resveratrol inclusion complex

#### 3.3.1 Moisture, yield, and resveratrol content

The systematic evaluation of processing parameters revealed critical insights into the optimization of resveratrol encapsulation. Experimental data demonstrated pronounced differences in both resveratrol retention and moisture dynamics depending on the molar ratios of resveratrol to HP-β-CD and the selected drying methodologies. As shown in Table 2, increasing the molar ratio from 1:1 to 1:2 significantly enhanced resveratrol content across all drying methods (*P* < 0.05). This aligns with prior findings that higher cyclodextrin ratios improve inclusion complex stability and entrapment efficiency (Aiassa et al., 2023; Saokham et al., 2018). Concurrently, moisture content in inclusion complexes varied markedly (*P* < 0.05) depending on the drying technique. The hygroscopic nature of HP-β-CD in 1:2 ratio complexes led to elevated moisture retention (Yang et al., 2022). Solvent evaporation emerged as the most effective method for moisture reduction, attributed to its prolonged drying phase enabling thorough dehydration.

**TABLE 2:** Assay test, yield percentage, and moisture content result.

Formula	RES content (%)	Yield (%)	Moisture content (%)
1:1			
RHSD	90.96 ± 0.95	71.50 ±1 .02	4.04 ±0.38 *
RHFD	93.48 ± 0.65	77.86 ±1 .17	3.19 ±0.45
RHSE	92.80 ± 0.40	87.83 ±0.99 *	2.35 ±0.67
1:2			
RHSD	97.65 ±1.49 *	85.43 ±1 .85*	4.39 ±1 .21*
RHFD	100.40 ± 0.89	87.83 ±1 .96	3.30 ±0.59
RHSE	99.25 ± 0.77	88.20 ±0.99	2.55 ±1.20

*Data are mean values (n = 3). Statistical analysis was performed using ANOVA, followed by Tukey’s test (p < 0.05). For each parameter (RES, content, Yield, Moisture Content) and within each molar ratio group (1:1 or 1:2), values marked with * are significantly different from values without * in the same column and ratio group.

While solvent evaporation demonstrated superior resveratrol recovery and moisture content, spray drying exhibited critical limitations linked to thermal instability. Specifically, spray-dried 1:1 and 1:2 samples showed the lowest resveratrol content, primarily due to thermal degradation during high-temperature processing (Buljeta et al., 2022). Lyophilized powders 1:2 retained significantly higher resveratrol content (100.40 ± 0.89%) compared to spray-dried counterparts (97.65% ± 1.49%), with Wilkowska et al. (2016) reporting a 73% loss of total polyphenols during spray drying (Wilkowska et al., 2016). This degradation involves the structural breakdown of resveratrol, including functional group loss and decomposition into inactive by-products (Ling et al., 2022). The superior recovery efficiency of solvent evaporation than spray drying stems from its controlled isothermal drying, which minimizes thermal degradation by maintaining low processing temperatures (<100 °C). This method aligns with Flory-Huggins thermodynamics (χ < 0.5), favoring stable resveratrol-cyclodextrin complexation through gradual solvent removal and homogeneous moisture depletion (Mura, 2015; Zhang et al., 2015). The absence of shear stress and rapid phase transitions preserves resveratrol’s labile structure, consistent with glass transition (*Tg*) theory, thereby optimizing recovery (Cash et al., 2013).

#### 3.3.2 Saturated solubility analysis

Resveratrol complexed powders were prepared by different drying methods which impacted the dry product characteristics. As shown in Table 3, all complexed powders (RHSD, RHFD, RHSE) gave significant solubility enhancement results compared with pure resveratrol. These results proved that the complexation of RES with HP-β-CD improved resveratrol aqueous solubility. Resveratrol has low water solubility of 0.548 mg/mL, whereas, its solubility increased up to 89-fold after complexation. In all the formulae, it can be seen that different ratios affect the solubility of the saturated complexation powder, with a 1:2 ratio showing a higher increase in solubility. As the ratio of cyclodextrin to guest molecule (drug) increases, more cyclodextrin molecules are available to form inclusion complexes with drug molecules (Yang et al., 2022). The result is an increase in the likelihood that each drug molecule will be encapsulated and an increase in the saturation solubility of resveratrol. The enhancement may be due to the presence of HP-β-CDs as a complexing agent and it helps to decrease the surface tension between the drug and the aqueous phase (Zafar et al., 2022). Additionally, drying treatment also observed to affect the saturated solubility of the complex. RHSD complexation was found to have higher saturated solubility among the three types of drying methods. Enhanced saturated solubility observed in the spray-dried (RHSD) complex can be attributed to the amorphization of resveratrol during the spray drying process. The amorphization process develops resveratrol into a higher-energy amorphous state that is thermodynamically more soluble while preventing the reorganization into crystalline forms (Escobar-Avello et al., 2021).

**TABLE 3:** Saturated solubility of inclusion complex in various methods.

Formula	Saturated solubility (mg/mL)
RES	0.548 ±0.016 ^a^
1:1	
RHSD	45.93 ±0.924 ^b^
RHFD	27.61 ±0.426 ^d^
RHSE	28.06 ±0.064 ^d^
1:2	
RHSD	48.85 ±0.388 ^c^
RHFD	28.19 ±0.052 ^d^
RHSE	36.86 ±0.527 ^e^

*Data are mean values (n = 3). Data followed with different letters are significantly different by ANOVA followed by Tukey’s test at a 5% level of significance (p < 0.05).

^a–e^These letters indicate statistically significant differences between groups based on one-way ANOVA followed by Tukey’s post-hoc test at a significance level of p < 0.05. Values sharing the same letter are not significantly different from each other, while values with different letters are statistically different.

#### 3.3.3 Antioxidant activity

**FIGURE 5:**
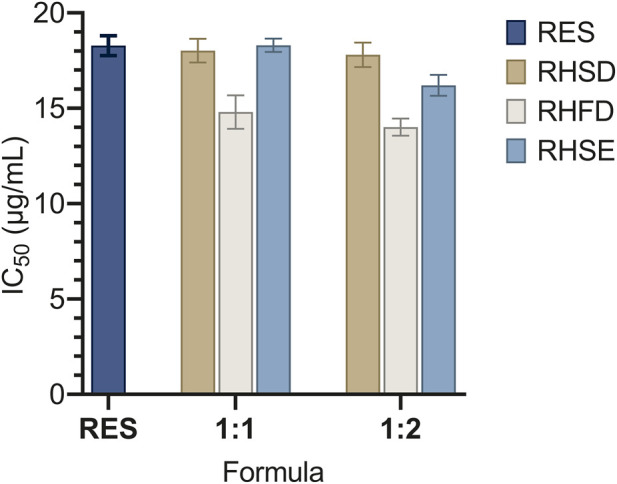
Comparison of IC_50_ values of resveratrol and its inclusion complexes preared by different methods at, molar ratios of 1:1 and 1:2.

The antioxidant activity of resveratrol and its inclusion complexes was evaluated based on IC_50_ values, as shown in Figure 5. A lower IC_50_ value indicates stronger antioxidant activity (Zhang et al., 2024). The results demonstrate that all inclusion complexes exhibited antioxidant activity compared to pure resveratrol, with variations depending on the preparation method and the molar ratio of resveratrol to HP-β-CD. All inclusion complexes exhibited comparable or improved antioxidant activity relative to pure resveratrol (18.28 μ g/mL), with freeze-dried (RHFD) complexes showing the most pronounced enhancement. Specifically, RHFD 1:2 and RHFD 1:1 achieved IC_50_ values of 14.01 μg/mL and 14.80 μg/mL, respectively, both significantly lower than pure resveratrol (*p < 0.05*). This improvement is attributed to freeze drying’s low-temperature processing, which preserves resveratrol bioactive trans-isomer by avoiding thermal degradation. The intact trans-isomer retains superior free radical scavenging capacity, while HP-β-CD complexation enhances molecular dispersion, increasing resveratrol accessibility to reactive oxygen species (Buljeta et al., 2022). Detailed results on the antioxidant activity and IC_50_ calculations are provided in Supplementary Appendix 3.

In contrast, spray-dried (RHSD) and solvent-evaporated (RHSE) complexes showed limited improvement. RHSD 1:2 and RHSD 1:1 had IC_50_ values of 17.80 μg/mL and 18.02 μg/mL, respectively statistically similar to pure resveratrol (*p > 0.05*). Similarly, RHSE 1:2 and RHSE 1:1 yielded moderate values (16.20 μg/mL and 17.30 μg/mL). A possible explanation for this is the thermal exposure involved in both solvent evaporation and spray drying, which may induce partial degradation of resveratrol, consequently reducing its antioxidant potential. Previous studies have reported that resveratrol is highly sensitive to heat, leading to the conversion of trans-resveratrol to its cis form, which has lower antioxidant activity (Zhang et al., 2024). These findings underscore that while HP-β-CD complexation generally stabilizes resveratrol, the preparation method critically determines its antioxidant efficacy. Freeze drying excels by preserving molecular integrity and optimizing dispersion, whereas spray drying and solvent evaporation compromise bioactivity due to heat-induced isomerization.

### 3.4 Physichochemical characteristics of optimum inclusion complex

#### 3.4.1 Functional group analysis

**FIGURE 6:**
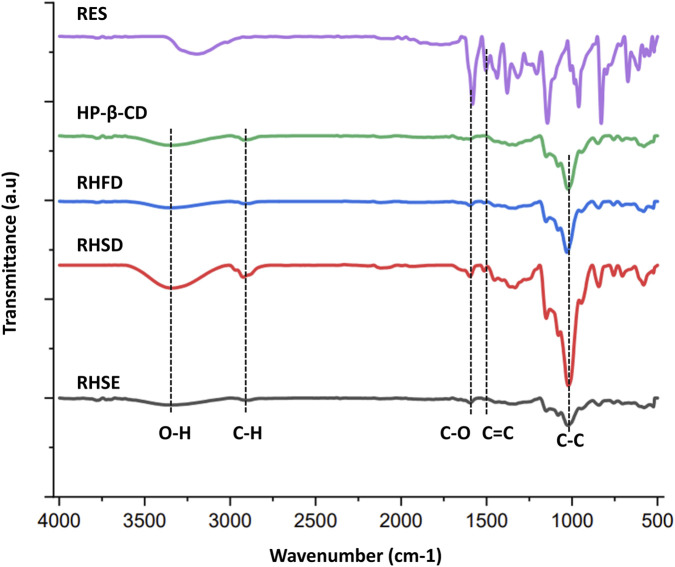
Spectra of resveratrol, HP-β-CD, RHSD, RHFD, and RHSE (1:2).

The infrared spectra illustrate how the active ingredient interacts with the encapsulation carrier. Figure 6 depicts the IR patterns for resveratrol and inclusion complex. All spectra exhibit a broad absorption band between 3000 and 3500 cm^-1^, attributed to the stretching vibrations of hydroxyl (-OH) groups (Lodagekar et al., 2019). Resveratrol exhibits a characteristic absorption band at 1503.85 cm^-1^, corresponding to the C=C stretching vibration of the olefinic bond in its aromatic structure (Silva et al., 2020; Yang T. X. et al., 2024). Moreover, HP-β-CD displays characteristic absorption bands at 3343.93 cm^-1^ (H-O-H stretching vibrations), 2920.04 cm^-1^ (aliphatic C-H stretching), 1633.95 cm^-1^ (O-H deformation vibration), 1599.72 cm^-1^ (C-O stretching), 1330.26 cm^-1^ (C–H bending vibration), 943.82 cm^-1^ (C–H bending vibrations in methyl (CH_3_) groups), 1150 cm^-1^ (C-O stretching vibration), and 1080 cm^-1^ (C-O stretching vibration) (Berta et al., 2021; Wang et al., 2022; Wang et al., 2020).

The IR spectra of the RHSD, RHFD, and RHSE (1:2) inclusion complexes are identical to that of HP-β-CD. Furthermore, the stability of the C-C (1150.56 cm^-1^) and C-O (1080.98 cm^-1^) bonds in HP-β-CD across all inclusion complexes indicates that the fundamental cyclodextrin structure remains intact during complexation. Compared to other drying methods, the increased intensity of the C-O-C peak observed in RHSD suggests stronger molecular interactions between resveratrol and HP-β-CD. Additionally, new peaks emerging at 1513.17 cm^-1^, 1513.71 cm^-1^, and 1511.70 cm^-1^ in the complexes can be attributed to conformational changes of resveratrol upon encapsulation, affecting the vibrations of olefinic double bonds. A decrease in the intensity of the trans-olefinic H-C=C-H bending peak further confirms the entrapment of resveratrol within the cyclodextrin structure. When guest molecules are encapsulated in the host molecule’s cavity, the vibration of the guest functional groups becomes restricted, resulting in lower-intensity peaks (Buljeta et al., 2022; Wilkowska et al., 2016). Furthermore, the absorption peak corresponding to O-H stretching shifted to 3341 cm^-1^ (RHSD), 3337.68 cm^-1^ (RHFD), and 3346.32 cm^-1^ (RHSE) compared to pure resveratrol. These shifts indicate the formation of hydrogen bonds between the hydroxyl (-OH) groups of resveratrol and HP-β-CD, confirming the successful inclusion of complex formation (Zhou et al., 2012). The spectral changes observed in these drying methods affirm that resveratrol has been effectively encapsulated within the cyclodextrin cavity.

FTIR spectra also revealed that the RHSD spectrum exhibits a broad OH stretching peak at 3341 cm^-1^, indicating stronger hydrogen bonding, reduced crystallinity, and enhanced interaction with water molecules. This phenomenon demonstrates significant molecular interactions between resveratrol and its excipients. Moreover, the C-O-C stretching region (1000–1200 cm^-1^) primarily associated with ether linkages in HP-β-CD plays a critical role in complex formation (Hecker et al., 2022). The higher intensity observed in the RHSD sample indicates enhanced molecular interactions between resveratrol and HP-β-CD, suggesting stronger hydrogen bonding and more efficient complex formation.

#### 3.4.2 Crystallinity properties (XRD)

**FIGURE 7:**
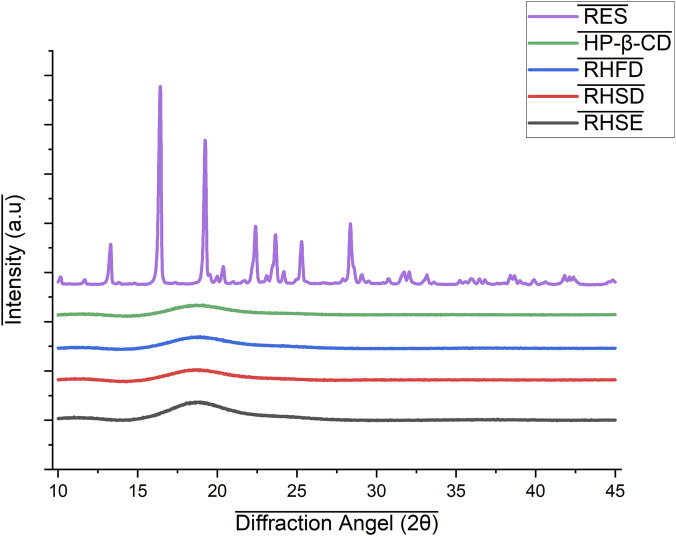
XRD of resveratrol and inclusion complex 1:2.

X-ray diffraction analysis (XRD) shows that the drying method significantly impacts the crystallinity level of inclusion complex. Resveratrol diffractogram at Figure 7. Shows strong crystalline structure characteristics with sharp diffraction peaks at 10.16°, 16.43°, 19.24°, and 23.64°, corresponding to the monoclinic polymorphic form (Form I) of resveratrol (Peng et al., 2019). This assignment is consistent with previous studies reporting that pure trans-resveratrol exhibits distinct reflections at 2θ = 6°–24°, characteristic of its monoclinic crystalline structure (Wang et al., 2022). On the other hand, HP-β-CD diffractogram possess broad, non-sharp diffraction band with a prominent peak around 18.7°, indicating amorphous properties due to the irregularity of the cyclodextrin molecular arrangement (He et al., 2019). After complex formation, the diffraction pattern of each preparation method shows significant changes compared to pure resveratrol and HP-β-CD, which indicates the occurrence of complexation and changes in the crystallinity of resveratrol.

In the complex prepared by the spray drying method (RHSD), the crystalline peaks of resveratrol disappeared, and only a dominant broad peak at 18.46° observed, which was in line with the amorphous pattern of HP-β-CD. This confirms that resveratrol has transitioned to the amorphous phase caused by the rapid spray drying process. The solvent’s high cooling rate and instant evaporation during spray drying prevent the resveratrol molecules from reorganizing into a crystalline structure, making the resulting complex amorphous. This phenomenon aligns with the report by Yang et al. (2024), which states that spray drying often produces amorphous systems because rapid drying inhibits crystallization.

In the freeze drying (RHFD) complex, the disappearance of the original resveratrol peak and the appearance of new peaks at 18.65° and 23.4° indicate a semi-crystalline phase that occurs due to the partial reorganization of resveratrol and HP-β-CD during sublimation. Roos (2021) states that freeze drying can cause partial recrystallization due to prolonged exposure to low temperatures. A similar pattern is also seen in the RHSE complex, which peaks at 18.81° and 24.05°. The slow evaporation process allows resveratrol and HP-β-CD to arrange themselves more regularly, in line with the theory of nucleation and crystal growth, which states that a low evaporation rate supports the formation of a more stable complex (Zhou et al., 2012).

The XRD analysis demonstrates that spray drying is the most effective technique for producing amorphous complexes, which significantly enhance the aqueous solubility of resveratrol. In contrast, freeze drying and solvent evaporation yield semi-crystalline structures that contribute to improved physicochemical stability while still offering enhanced solubility. Consequently, the selection of the preparation method should be strategically aligned with the formulation’s objective.

#### 3.4.3 Thermal properties study

**FIGURE 8:**
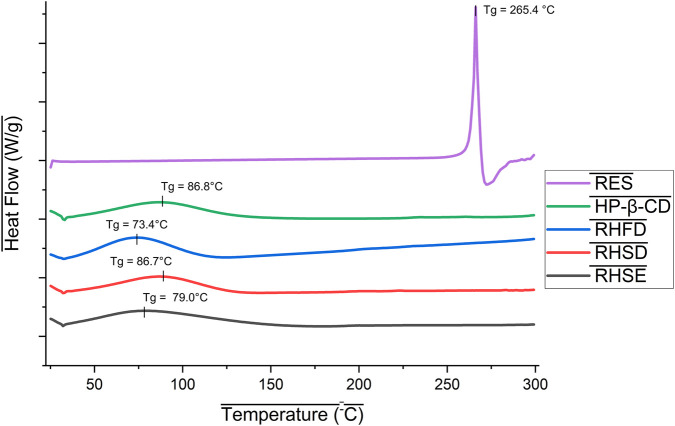
DSC thermogram of resveratrol and inclusion complex 1:2.

The thermal characteristics of resveratrol, HP-β-CD, and the inclusion complexes RHSD, RHFD, and RHSE were analyzed using Differential Scanning Calorimetry (DSC). Figure 8 shows DSC thermogram of pure resveratrol shows a sharp endothermic peak at 265.4 °C (322.7 J/g). The high enthalpy value and peak sharpness indicate high crystallinity and sample purity, consistent with the resveratrol crystalline structure reported in the literature (Yang T. X. et al., 2024). In contrast, HP-β-CD displays an amorphous nature, indicated by a broad endothermic peak at 86.8 °C (ΔH = 171.2 J/g), corresponding to the combined processes of water desorption and glass transition within the amorphous phase (Hao et al., 2021; Souza et al., 2022).

The disappearance of the resveratrol characteristic endothermic peak at 265.4 °C in the RHSD, RHFD, and RHSE samples indicates that resveratrol has undergone a transformation from the crystalline phase to the amorphous phase due to the intercalation of resveratrol molecules into the hydrophobic cavity of HP-β-CD (Souza et al., 2022). This transformation is consistent with the inclusion complex mechanism, where the disruption of the crystal structure of resveratrol through molecular interaction with the cyclodextrin matrix leads to the loss of resveratrol crystallinity, further supported by the observed decrease in endothermic peaks of the complexes (Nishihira et al., 2016). Specifically, Figure 8 shows the endothermic peaks of HP-β-CD shifted from 86.8 °C (original sample) to 86.7 °C (RHSD), 73.4 °C (RHFD), and 79.0 °C (RHSE), with enthalpy changes of 159.5 J/g, 187.6 J/g, and 185.4 J/g, respectively.

The shift in endothermic peaks reflects the modification of molecular mobility and thermal stability of the system due to the interaction of resveratrol with HP-β-CD. The decreased trend of endothermic peak in RHFD (73.4 °C) is associated with the plastic effect of residual moisture or disruption of hydrogen bonds between HP-β-CD molecules post-freeze-drying process (Buljeta et al., 2022). Meanwhile, the enthalpy variation shows the difference in the level of energy required to weaken intermolecular interactions in complex systems, depending on the method of preparation (spray drying, freeze-drying, or solvent evaporation). These results are in line with the theory that the formation of inclusion complexes modifies the thermodynamic properties of amorphous matrices through the redistribution of non-covalent bonds (Wilkowska et al., 2016).

#### 3.4.4 Morphology analysis

**FIGURE 9:**
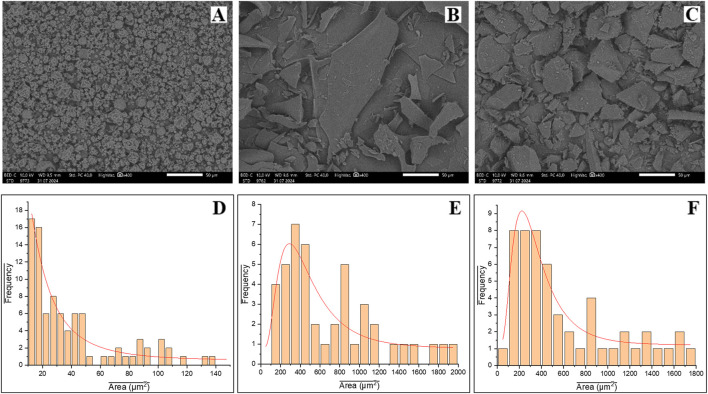
SEM images of **(A)** RHSD; **(B)** RHFD; **(C)** RHSE; ImageJ Quantitative Analysis of Particle Size **(D)** RHSD; **(E)** RHFD; **(F)** RHSE.

The scanning electron microscopy (SEM) images illustrate the morphological differences in resveratrol-cyclodextrin complexes formed by various drying methods, as depicted in Figure 9. The drying technique significantly affects the particle inclusion complex morphology, which influences the complex’s physical properties and performance (Berta et al., 2021; Lodagekar et al., 2019). As seen in the micrograph images at ×400 magnification in Figure 9A, rapid drying in spray drying leads to the formation of spherical particles that exhibit an amorphous structure with a high surface area. The smooth surface indicates that resveratrol is well encapsulated within the cyclodextrin, with minimal crystallization occurring during the rapid drying process. This is consistent with previous studies, which have demonstrated that spray drying promotes the amorphization of drug-cyclodextrin complexes, leading to enhanced solubility of resveratrol (Wilkowska et al., 2016). Additionally, the rapid drying process prevents crystal formation due to insufficient nucleation and crystal growth time.

In comparison, the inclusion complexes obtained through lyophilization, observed in Figure 9B at ×400 magnification, exhibit a crystalline lamellate morphology, consistent with earlier reports (Buljeta et al., 2022). The formation of thin, plate-like structures indicates that the inclusion complex retains a significant crystalline component (Zhou et al., 2012), suggesting that lyophilization influences the structural arrangement of the complex, resulting in a more ordered, layered crystalline form that differs from the other drying methods. Similarly, the solvent evaporation method, shown in Figure 9C at ×400 magnification, reveals a more fragmented morphology with sharp edges and smooth surfaces. This indicates that the drying process leads to the formation of a crystalline structure with well-defined particle boundaries, involving nucleation and crystal growth. During solvent evaporation, the molecules have sufficient time to arrange themselves in an orderly pattern, facilitating gradual crystallization and resulting in a well-defined crystalline morphology (Zhou et al., 2012).

In addition to these morphological observations using SEM, quantitative analysis with ImageJ in Figures 9D–F confirmed the differences in particle size between the drying methods. Spray drying produced particles with an average area of 39.98 µm^2^, forming spherical, smooth, and amorphous particles. The rapid drying process prevents crystal formation, which is consistent with the theory that rapid drying minimizes the time available for nucleation and crystal growth (Yang et al., 2022). On the other hand, freeze drying produced larger particles with an average area of 694.86 µm^2^, consistent with a layered crystalline morphology. The slower sublimation process in freeze drying allows for more controlled recrystallization, resulting in a more ordered structure (Nowak and Jakubczyk, 2020). Solvent evaporation showed particles with an average area of 611.65 µm^2^, with a more defined crystalline morphology, as the gradual evaporation of the solvent allows molecules to arrange themselves in a regular pattern (Zhou et al., 2012). These SEM and ImageJ analysis results demonstrate that spray drying produces smaller and amorphous particles, while freeze drying and solvent evaporation yield larger, more crystalline particles. These differences in particle morphology directly influence the solubility and release profiles of the complexes, with amorphous preparations typically showing better solubility and faster drug release. In contrast, crystalline preparations offer higher stability but slower release.

### 3.5 Drug release and kinetics models

**FIGURE 10:**
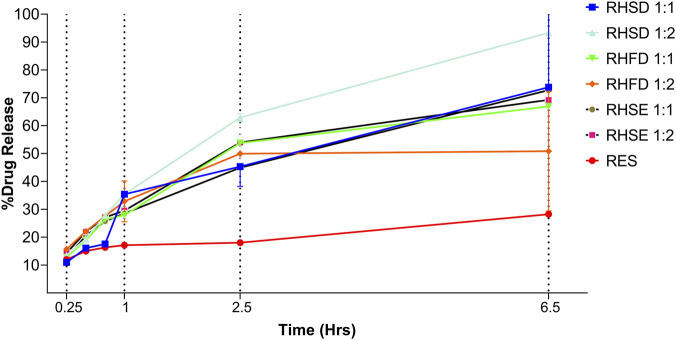
*In vitro* release profiles of resveratrol and inclusion complex in various drying methods.

The release study revealed that the amount of drug release differed significantly between resveratrol complex and pure resveratrol. Based on the study’s results, the percentage of inclusion complex release using different drying methods shows a significant difference compared to pure resveratrol. As observed in Figure 10, inclusion complexes with a 1:2 ratio possess a faster release rate due to hydrophilicity improvement compared to a 1:1 ratio inclusion complex. Specifically, RHSD 1:2 achieved 93.33% ± 0.010% drug release after 6.5 h, significantly higher (*p < 0.05*) than RHSD 1:1 (73.79% ± 0.03%). This improvement is attributed to the amorphous structure of spray-dried particles, which increases hydrophilicity and reduces energy barriers for dissolves. According to the Gibbs Free Energy theory, the disordered amorphous state possesses higher entropy than crystalline forms, enabling faster solubility and drug release a critical factor for formulations requiring rapid bioavailability (Baghel et al., 2016).

In contrast, RHFD and RHSE complexes showed slower release rates due to their retained crystallinity. For instance, RHFD 1:1 and RHSE 1:1 released only 58.7% ± 0.003% and 69.2% ± 0.007% (*p < 0.05* vs. RHSD), respectively, even at a 1:2 ratio, their performance remained limited (RHFD 1:2 69.2% ± 0.090%; RHSE 1:2 72.9% ± 0.34%). The crystalline morphology in these methods requires more energy to disrupt intermolecular bonds, resulting in delayed release. Notably, the differences between RHFD and RHSE were not statistically significant (*p > 0.05*), highlighting the dominance of spray drying in optimizing release kinetics. Detailed data and calculations regarding the release profiles are provided in Supplementary Appendix 4.

The release kinetics of the resveratrol complexes are significantly influenced by the drying method, which determines both the matrix structure and the release mechanism. The data presented in Table 4 indicate that the drying method impacts the release mechanism, which involves both diffusion and more complex mechanisms. From the table, shows that the Korsmeyer-Peppas model is the best-fitting model for the RHSD 1:1 and RHSD 1:2 inclusion complexes, with diffusion exponent values *n* greater than 0.45. Specifically, for RHSD 1:2, the Korsmeyer-Peppas model shows an R^2^ value of 0.989 and a diffusion exponent (*n*) of 0.615, suggesting a non-Fickian (anomalous) release mechanism. This indicates that the release of resveratrol from the spray-dried complex involves both diffusion and polymer relaxation, as described by Sakran et al. (2024) the high Model Selection Criterion (MSC) value of 22.5 for this sample further supports that the Korsmeyer-Peppas model is the most appropriate for describing the release profile, as the MSC accounts for the goodness of fit and model complexity.

**TABLE 4:** Mathematical models of drug release kinetics: application to resveratrol- HP-β-CD inclusion complexes.

Sample	Zero order		First order		Higuchi		Krosmeyer-peppas		
	R^2^	MSC	R^2^	MSC	R^2^	MSC	R^2^	n	MSC
RES	0.938	3.02	0.847	1.38	0.980	12.25	0.981	0.257	12.91
RHSD 1:1	0.913	2.10	0.739	0.71	0.941	3.98	0.963	0.601	6.51
RHSD 1:2	0.929	2.62	0.764	0.81	0.987	18.98	0.989	0.615	22.5
RHFD 1:1	0.858	1.21	0.725	0.66	0.973	9.02	0.951	0.525	4.85
RHFD 1:2	0.889	1.60	0.741	0.71	0.974	9.37	0.967	0.398	7.33
RHSE 1:1	0.920	2.30	0.756	0.77	0.988	20.58	0.987	0.530	18.96
RHSE 1:2	0.881	1.48	0.749	0.75	0.982	13.64	0.965	0.493	6.89

In contrast, complexes RHFD and RHSE adhered to the Higuchi model, as evidenced by high R^2^ values (0.973 for RHFD 1:1 and 0.988 for RHSE 1:1) and the *n* values closer to 0.5. These results suggest that the release of resveratrol in these complexes primarily follows a diffusion-controlled release, where the drug is released from the matrix over time through simple diffusion. The high MSC values for RHFD and RHSE further validate that the Higuchi model fits the release profile well, reflecting the stable and structured matrix of these complexes.

Furthermore, these results highlight the importance of considering drying methods when designing drug delivery systems. Tailoring matrix properties through different drying techniques can influence the rate and mechanism of drug release, thereby improving the therapeutic efficacy of the formulation. The combination of R^2^, *n*, and MSC provides a comprehensive approach to model selection, ensuring that the most suitable model is chosen for describing the release kinetics of bioactive compounds in drug delivery systems (Culas et al., 2024).

## 4 Conclusion

This study successfully developed resveratrol–HP-β-CD inclusion complexes with high stability (K = 5278 M^-1^), where the 1:2 M ratio demonstrated optimal performance across all preparation methods. Among all complexes, spray drying yielded amorphous particles with the most excellent solubility enhancement (89-fold increase to 48.85 mg/mL), whereas freeze drying best preserved antioxidant activity (IC_50_ = 14.01 μg/mL). Characterization by FTIR confirmed the formation of inclusion complexes through the attenuation and shifting of characteristic resveratrol peaks, indicating strong host–guest interactions within the HP-β-CD cavity. XRD and DSC analyses revealed distinct differences in crystallinity. RHSD produced a fully amorphous structure, whereas RHFD and RHSE retained semi-crystalline characteristics. SEM observations supported these findings, showing spherical, uniform particles for RHSD and irregular morphologies for RHFD and RHSE. Release kinetics further demonstrated that RHSD followed non-Fickian transport (n = 0.615) with 93.33% cumulative drug release. At the same time, RHFD and RHSE exhibited diffusion-controlled release profiles. These results show that spray drying is the most effective method for improving solubility and dissolution by forming amorphous complexes. In contrast, freeze drying is advantageous for maintaining resveratrol’s antioxidant stability. The integration of physicochemical and kinetic analyses provides clear guidance for selecting drying techniques to optimize HP-β-CD-based resveratrol formulations for pharmaceutical and nutraceutical applications.

## Data Availability

The original contributions presented in the study are included in the article/Supplementary Material, further inquiries can be directed to the corresponding authors.
