# Supplementary material for: Mechanistic insights into drying methods: how they govern the structure and bioactivity of resveratrol-HP-β-CD inclusion complexes
Source: Front Chem. 2025 Dec 19;13:1692674. doi: 10.3389/fchem.2025.1692674 (PMC12757797; doi:10.3389/fchem.2025.1692674)
Supplement: Supplementary file 1 [file DataSheet1.docx]

# Supplementary Materials

Appendix 1 Resveratrol Standart Graph

| Concentration (ppm) | UV.Vis Area | | | Avg | SD | RSD |
| --- | --- | --- | --- | --- | --- | --- |
|  | 1 | 2 | 3 |  |  |  |
| 2,0 | 0,278 | 0,278 | 0,277 | 0,278 | 0,001 | 0,21% |
| 2,5 | 0,368 | 0,367 | 0,369 | 0,368 | 0,001 | 0,27% |
| 3 | 0,444 | 0,445 | 0,444 | 0,444 | 0,001 | 0,13% |
| 3,5 | 0,529 | 0,529 | 0,530 | 0,529 | 0,001 | 0,11% |
| 4 | 0,598 | 0,599 | 0,598 | 0,598 | 0,001 | 0,10% |
| 5 | 0,767 | 0,766 | 0,768 | 0,767 | 0,001 | 0,13% |
| 5,5 | 0,843 | 0,844 | 0,843 | 0,843 | 0,001 | 0,07% |

Slope = 0,1604

Intercept = -0,0378

R^2^ = 0,9995

R = 0,9997

Appendix 1 The phase-solubility data of resveratrol and HP-β-CD

| HP-β-CD (mM) | RES (mM) | | | Avg | SD | RSD |
| --- | --- | --- | --- | --- | --- | --- |
|  | 1 | 2 | 3 |  |  |  |
| 0 | 0,270 | 0,269 | 0,271 | 0,270 | 0,001 | 0,42% |
| 2 | 1,751 | 1,761 | 1,761 | 1,758 | 0,006 | 0,33% |
| 5 | 3,547 | 3,553 | 3,547 | 3,549 | 0,003 | 0,09% |
| 10 | 6,899 | 6,951 | 6,912 | 6,921 | 0,027 | 0,39% |
| 15 | 10,054 | 10,036 | 10,073 | 10,054 | 0,019 | 0,19% |

Slope = 0,6498

Intercept = 0,3515

R^2^ = 0,9996

R = 0,9997

$$Kc=\frac{Slope}{S0 (1-Slope)}$$

$$Kc=\frac{0,6498}{0,3515 (1-0,6498)}$$

$$Kc=5,278mM^{-1} \cong5278M^{-1}$$

Appendix 2 Job’s plot different molar ratios of resveratrol and HP-β-CD from absorbance 306 nm measurements

| Res : HP-β-CD | RES (ppm) | | | Avg | SD | RSD |
| --- | --- | --- | --- | --- | --- | --- |
|  | 1 | 2 | 3 |  |  |  |
| 0,1 | 2,136 | 2,136 | 2,134 | 2,135 | 0,001 | 0,050% |
| 0,2 | 3,481 | 3,482 | 3,481 | 3,482 | 0,001 | 0,017% |
| 0,3 | 4,743 | 4,743 | 4,743 | 4,743 | 0,000 | 0,002% |
| 0,4 | 4,936 | 4,936 | 4,936 | 4,936 | 0,000 | 0,000% |
| 0,5 | 4,249 | 4,249 | 4,249 | 4,249 | 0,000 | 0,007% |
| 0,6 | 3,837 | 3,838 | 3,837 | 3,838 | 0,000 | 0,012% |
| 0,7 | 3,148 | 3,149 | 3,148 | 3,148 | 0,001 | 0,023% |
| 0,8 | 2,658 | 2,660 | 2,664 | 2,661 | 0,003 | 0,116% |
| 0,9 | 2,007 | 2,007 | 2,007 | 2,007 | 0,000 | 0,000% |

Appendix 3 Comparison of IC₅₀ values of resveratrol and its inclusion complexes preared by different methods at , molar ratios of 1:1 and 1:2.

| Formula | IC50 (µg/mL) | | | Avg | SD | RSD |
| --- | --- | --- | --- | --- | --- | --- |
|  | 1 | 2 | 3 |  |  |  |
| RES | 18,260 | 18,290 | 18,280 | 18,277 | 0,015 | 0,08% |
| 1:1 | | | | | | |
| RHSD | 18,050 | 18,010 | 18,000 | 18,020 | 0,026 | 0,15% |
| RHFD | 14,800 | 14,900 | 14,800 | 14,833 | 0,058 | 0,39% |
| RHSE | 18,310 | 18,310 | 18,290 | 18,303 | 0,012 | 0,06% |
| 1:2 | | | | | | |
| RHSD | 17,810 | 17,830 | 17,780 | 17,807 | 0,025 | 0,14% |
| RHFD | 14,010 | 14,020 | 14,000 | 14,010 | 0,010 | 0,07% |
| RHSE | 16,240 | 16,230 | 16,140 | 16,203 | 0,055 | 0,34% |

Appendix 4 HPLC Calibration Curve for Resveratrol

| Concentration (ppm) | Area | | | Avg | SD | RSD (%) |
| --- | --- | --- | --- | --- | --- | --- |
|  | 1 | 2 | 3 |  |  |  |
| **0.5** | 58.415 | 60.146 | 59.348 | 59.303 | 866 | 1,46% |
| **2** | 184.655 | 178.816 | 179.296 | 180.922 | 3.241 | 1,79% |
| **5** | 523.270 | 525.890 | 519.909 | 523.023 | 2.998 | 0,57% |
| **10** | 1.261.830 | 1.277.530 | 1.275.700 | 1.271.687 | 8.585 | 0,68% |
| **15** | 1.935.150 | 1.957.776 | 1.949.495 | 1.947.474 | 11.448 | 0,59% |
| **25** | 3.177.035 | 3.199.586 | 3.166.921 | 3.181.181 | 16.722 | 0,53% |
| **50** | 6.215.120 | 6.433.354 | 6.394.997 | 6.347.824 | 116.514 | 1,84% |

Slope = 128550,0284

Intercept = -50457,1737

R^2^ = 0,9995

R = 0,9997

- In vitro dissolution profiles of resveratrol and inclusion complex in various drying methods

Table 1 Resveratrol

| RES | | | | | |
| --- | --- | --- | --- | --- | --- |
| Time (Hrs) | Drug Release (%) | | | Avg (%) | SD |
|  | 1 | 2 | 3 |  |  |
| 0,25 | 11,5 | 13,9 | 10,6 | 12,00 | 1,71 |
| 0,5 | 15 | 15,4 | 14,7 | 15,03 | 0,35 |
| 0,75 | 16,4 | 16,3 | 16,3 | 16,33 | 0,06 |
| 1 | 17,3 | 17,2 | 16,9 | 17,13 | 0,21 |
| 2,5 | 18,1 | 17,8 | 18,2 | 18,03 | 0,21 |
| 6,5 | 28,1 | 27,9 | 28,7 | 28,23 | 0,42 |

Table 2 RHSD 1:1

| RHSD 1:1 | | | | | |
| --- | --- | --- | --- | --- | --- |
| Time (Hrs) | Drug Release (%) | | | Avg (%) | SD |
|  | 1 | 2 | 3 |  |  |
| 0,25 | 10,8 | 11,1 | 11 | 10,97 | 0,15 |
| 0,5 | 16,4 | 16,1 | 15,8 | 16,10 | 0,30 |
| 0,75 | 17 | 18,1 | 17,6 | 17,57 | 0,55 |
| 1 | 34,8 | 35,5 | 35,9 | 35,40 | 0,56 |
| 2,5 | 49,2 | 49,6 | 37,1 | 45,30 | 7,10 |
| 6,5 | 89,9 | 88,1 | 43,4 | 73,80 | 26,34 |

Table 3 RHSD 1:2

| RHSD 1:2 | | | | | |
| --- | --- | --- | --- | --- | --- |
| Time (Hrs) | Drug Release (%) | | | Avg (%) | SD |
|  | 1 | 2 | 3 |  |  |
| 0,25 | 12,8 | 13,7 | 13,9 | 13,47 | 0,59 |
| 0,5 | 18,8 | 18,4 | 21,1 | 19,43 | 1,46 |
| 0,75 | 27,6 | 28,9 | 27 | 27,83 | 0,97 |
| 1 | 35,9 | 35 | 35,3 | 35,40 | 0,46 |
| 2,5 | 61,7 | 62,5 | 64,5 | 62,90 | 1,44 |
| 6,5 | 92,3 | 93,5 | 94,2 | 93,33 | 0,96 |

Table 4 RHFD 1:1

| RHFD 1:1 | | | | | |
| --- | --- | --- | --- | --- | --- |
| Time (Hrs) | Drug Release (%) | | | Avg (%) | SD |
|  | 1 | 2 | 3 |  |  |
| 0,25 | 12,9 | 12,8 | 12,9 | 12,87 | 0,06 |
| 0,5 | 18,9 | 19,1 | 18,8 | 18,93 | 0,15 |
| 0,75 | 26,9 | 26,2 | 26 | 26,37 | 0,47 |
| 1 | 28 | 27,8 | 27,9 | 27,90 | 0,10 |
| 2,5 | 53,8 | 53,3 | 54,1 | 53,73 | 0,40 |
| 6,5 | 66,9 | 66,7 | 67,1 | 66,90 | 0,20 |

Table 5 RHFD 1:2

| RHFD 1:2 | | | | | |
| --- | --- | --- | --- | --- | --- |
| Time (Hrs) | Drug Release (%) | | | Avg (%) | SD |
|  | 1 | 2 | 3 |  |  |
| 0,25 | 15,7 | 15,5 | 15,9 | 15,70 | 0,20 |
| 0,5 | 22,4 | 22 | 21,9 | 22,10 | 0,26 |
| 0,75 | 27,6 | 27,3 | 27,8 | 27,57 | 0,25 |
| 1 | 37 | 24,4 | 37,2 | 32,87 | 7,33 |
| 2,5 | 49,9 | 50,1 | 49,8 | 49,93 | 0,15 |
| 6,5 | 75,6 | 38,6 | 38,3 | 50,83 | 21,45 |

Table 6 RHSE 1:1

| RHSE 1:1 | | | | | |
| --- | --- | --- | --- | --- | --- |
| Time (Hrs) | Drug Release (%) | | | Avg (%) | SD |
|  | 1 | 2 | 3 |  |  |
| 0,25 | 13,7 | 12 | 12,3 | 12,67 | 0,91 |
| 0,5 | 22,3 | 19,2 | 19,9 | 20,47 | 1,63 |
| 0,75 | 27,5 | 24,6 | 25,2 | 25,77 | 1,53 |
| 1 | 32,5 | 26,6 | 26,4 | 28,50 | 3,47 |
| 2,5 | 53 | 40,1 | 41,7 | 44,93 | 7,03 |
| 6,5 | 73,1 | 72,1 | 73,1 | 72,77 | 0,58 |

Table 7 RHSE 1:2

| RHSE 1:2 | | | | | |
| --- | --- | --- | --- | --- | --- |
| Time (Hrs) | Drug Release (%) | | | Avg (%) | SD |
|  | 1 | 2 | 3 |  |  |
| 0,25 | 15 | 14,1 | 13,9 | 14,33 | 0,59 |
| 0,5 | 23 | 21,7 | 21 | 21,90 | 1,01 |
| 0,75 | 28,8 | 26,7 | 25,8 | 27,10 | 1,54 |
| 1 | 32,4 | 28,6 | 27,6 | 29,53 | 2,53 |
| 2,5 | 56,3 | 48,2 | 57,4 | 53,97 | 5,02 |
| 6,5 | 78,9 | 67,7 | 61,2 | 69,27 | 8,95 |

Table 8 Saturated Solubility Data

| **Formula** | **Saturated Solubility (mg/mL)** | | | Avg | SD | RSD |
| --- | --- | --- | --- | --- | --- | --- |
|  | 1 | 2 | 3 |  |  |  |
| **RES** | 0,531 | 0,551 | 0,562 | 0,548 | 0,0159 | 2,90% |
| **1:1** |  |  |  |  |  |  |
| **RHSD** | 46,210 | 46,687 | 44,902 | 45,933 | 0,9244 | 2,01% |
| **RHFD** | 27,830 | 27,127 | 27,895 | 27,617 | 0,4257 | 1,54% |
| **RHSE** | 27,988 | 28,086 | 28,110 | 28,062 | 0,0648 | 0,23% |
| **1:2** |  |  |  |  |  |  |
| **RHSD** | 48,795 | 49,264 | 48,494 | 48,851 | 0,3881 | 0,79% |
| **RHFD** | 28,133 | 28,237 | 28,201 | 28,190 | 0,0529 | 0,19% |
| **RHSE** | 36,390 | 36,769 | 37,431 | 36,863 | 0,5273 | 1,43% |
